# A Complete Repertory of the Tissue Remedies of Schüssler

**Published:** 1894-08

**Authors:** 


### BOOK NOTICES.

A Complete Repertory of the Tissue Remedies of Schüssler. By S. F. Shannon, M. D., Denver, Colorado. The Chain & Hardy Book, Stationery, and Art Company, 1894.

The Tissue Remedies of Schüssler have been edited by more ambitious writers than almost any other book. But of all these the best is that of Bœricke & Dewey, reviewed in this journal for May, 1893, page 300.

None, however, have ever turned the book into a repertory, though Bœricke & Dewey have added an excellent repertory to their edition. In the work now under review the Repertory is a series of chapters arranged according to the regions of the body. Each chapter again is arranged alphabetically. There can be no question of the need of a full repertory, especially since the expansion of the Schüssler remedies to their present dimensions.

With all due respect to Bœricke & Dewey's work we are of opinion that this Repertory forms a desirable companion to their work. We cordially recommend it.

